# Anthropogenic Effects of Coal Mining on Ecological Resources of the Central Indus Basin, Pakistan

**DOI:** 10.3390/ijerph17041255

**Published:** 2020-02-15

**Authors:** Abdul Jabbar Khan, Gulraiz Akhter, Hamza Farooq Gabriel, Muhammad Shahid

**Affiliations:** 1 Earth Sciences Department, Quaid-i-Azam University, Islamabad 44000, Pakistan; agulraiz@qau.edu.pk; 2 National University of Sciences and Technology (NUST), Islamabad 44000, Pakistan; hamza.gabriel@nice.nust.edu.pk (H.F.G.); m.shahid@nice.nust.edu.pk (M.S.)

**Keywords:** Coal mining, Potential Toxic Elements, Ecological resources, Central Indus basin, Contamination

## Abstract

Water is essential for life, agriculture, and industrialization; however, a rapid increase in population is constantly causing water scarcity and pollution in Pakistan. Mining activities produce the potential toxic element (PTE) accumulation, which lead to unnatural enrichment, ecological pollution, and environmental degradation. The ecological resources impeded by the PTEs cause serious abnormalities in the population through dermal contact, inhalation, and digestion. Mining induced anthropogenic activities are well-known causes of contamination of ecological resources. The produced effluents have drastic effects by changing the physical, chemical, and biological properties of the concerned resources. The Central Indus Basin is a well-known coal regime, where more than 160 mines are active at present. The samples that were collected from the mine water, groundwater, surface water, and the soil were analyzed by atomic absorption and elemental determination analysis (EDA) for an assessment of their quality and the presence of PTEs. The results were correlated with available quality standards, including the World Health Organization (WHO), National Standard of Drinking Water Quality (NSDWQ), World Wildlife Fund (WWF), and Sediment Quality Guidelines (SQGs). These analyses showed the noticeable anthropogenic concentration of PTEs, like iron, cadmium, sulphur, and copper, which can degrade the quality of resources in the Central Indus Basin and have adverse effects on human health. An excessive amount of acid mine drainage (AMD) draws attention to some suitable active or passive treatments for disposal from mines to avoid degradation of ecological resources in the Central Indus Basin of Pakistan.

## 1. Introduction

Coal resources are considered to be an essential conventional source of energy and a significant factor in developing the national economy. Surface mining of these resources has increased globally during the past few decades [1,2]. The exploitation of these resources can cause various adverse impacts. These adverse impacts become more challenging in the arid and semi-arid regions, which have a shortage of water resources. Pit dewatering occurs during the coal mining process, which might lead to dry spring and ecosystem degradation at the regional level. The environmental impacts in arid and semi-arid regions can be more severe if no precautionary measure is taken [3]. The environmental impacts include an imbalance between the demand and supply of water and water quality issues. The water quality degradation, irrigation water degradation, biodiversity, and soil quality issues are some other impacts of coal mining, which directly impact agricultural productivity and human health. Moreover, the regional hydrological cycle can be affected due to transformation changes of the surface water and groundwater, the increasing rate of infiltration, and the reduction in evaporation rate. The previous studies point out increment of the concentration of weathering solutes in streams near the mining areas [2,4]. The changes have been found in the concentration discharge relationship of the watershed before and after coal mining activities. Therefore, understanding the anthropogenic impacts of coal mining is necessary for better planning and management of ecological resources, particularly water resources.

At present, Pakistan is facing a challenge of energy crises. Therefore, coal is considered as one of the significant sources of energy production. Pakistan has the world’s largest lignitic coal reserves. There are over 90 billion tons of coal in Pakistan; 97% of coal reserves are lignitic, while only 3% have a maturity of sub-bituminous to bituminous [5,6]. In Pakistan, coal is used in sugar, steel, cement, and brick kilns, and domestic, and other small industries, including water and power development authorities. Moreover, recent projects, such as one belt one road, include developments of industrial zones in different cities of Pakistan, where coal will be used as the main source for energy production. 

Good reserves, promising investment-returns, and the rapid use of coal are threatening to environmental concerns in Pakistan. The environmental impacts of coal mining include air pollution, erosion, and quality issues for surface water and groundwater. Currently, degradation in water and soil resources is the main environmental hazard in Pakistan. The release of effluents causes the physical, chemical, and biological degradation of these resources; therefore, leachate is continuously causing contamination of local water and soil resources [7]. The Indus River is the major river of Pakistan, which fulfills the water requirements of all sectors, including domestic and industrial users. The coal mining activities are being carried out in different parts of the Indus Basin, which have affected the ecosystem of the basin and pose serious threats to the survival of humans and the environment, which needs to be evaluated. 

The conventional approaches generally include some simple un-integrated methods retrofitted by the statistical analysis. However, in this study, an integrated approach that was comprehensively based on experimental procedures has been introduced through the determination of geochemical background analysis for this region and comparison with globally available literatures. In this work, we develop the geochemical background of rock and soils along the geochemical indices and the pollution indices to judge the environmental risks of PTEs by comparing the available quality standard of soil and water. Although, limited studies on contamination of river sediments in Pakistan is available, however, none of the previous works provides credential information on the role of anthropogenic sources of PTEs, especially in the River Jhelum and its tributaries. Previous studies only provide some information about the PTEs on large riverine systems of this region on the basis of un-integrated traditional approaches, mainly relying on available data analysis and the simple statistical algorithms. 

The previous studies do not consider the geological knowledge, geographical constraints, basin morphology, and virgin ecological status of this region. The purpose of this study is to draw attention of the environmental agencies on national scale, and prognosticate the probable health impacts of anthropogenic activities. The main objectives of this research are (1) to develop geochemical background values; (2) to assess the contamination levels of PTEs by geochemical indices; and, (3) to draw the attention for urgent management of mining waste to address pollution issues. The novelty of this study is reflected by the assessment of geochemical backgrounds, which were unavailable for the typical Central Indus Basin, a part of Asia. Moreover, geochemical pollution indices have been exercised by using integrated approach to prognosticate the downstream transport of PTEs in the Central Indus Basin of Pakistan.

## 2. Materials and Methods 

### 2.1. Study Area 

**Figure 1:**
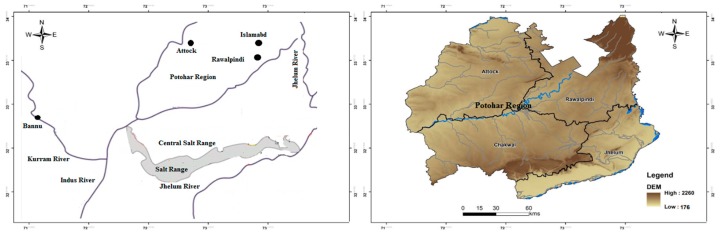
The location map of the Potohar region and salt range [10].

The study area lies in the salt range, which is a hill system in the Punjab province of Pakistan. The Salt Range, which is located between longitude 71°30′–73°30′ east and between latitude 32°23′–33°00′ north, forms a very prominent geomorphologic and ecological feature, as it is between the Thal Desert in the west and the Potohar Plateau in the northeast [8]. This range starts from the Potohar region and ends at the north side of the River Jhelum. The Jhelum River is one of the major tributaries of the Indus, flowing through this region, and it is considered as the central hydrological unit of the study area. Climatically, the area is characterized by low rainfall of about 50cm, and the months of July, August, and September are more rain-prone periods. Subtropical dry but evergreen shrubs are the main vegetation type [9]. Figure 1 presents the location map of the Potohar region and Salt Range.

**Figure 2:**
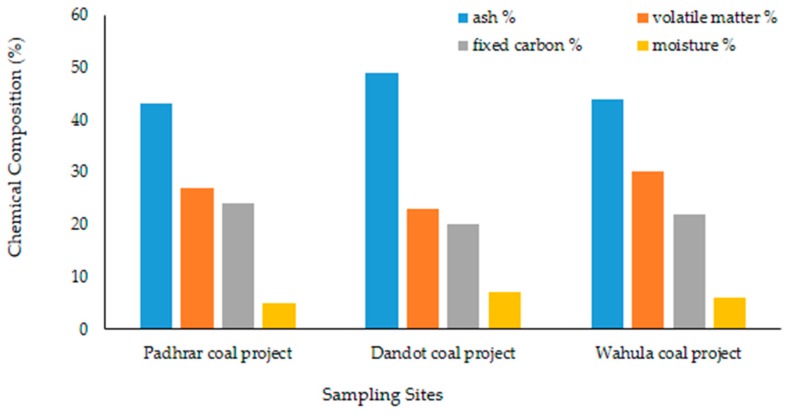
Graphical representation of the proximate analysis for coal of the Salt range from [11].

The Central Basin of the Indus River system is sedimentary, which represents Precambrian to recent stratigraphy, with large reserves of coal and other mineral resources. The coal geology is dominated by Permian and Paleocene stratigraphy in the central Indus basin. Permian coal is mined in the Chakwal Division. Similarly, tertiary coal is mined in the central and eastern Salt Range. Coal is found in the late Paleocene Patala Formation, in the Chakwal and Jhelum Districts. These coal horizons are friable, high in ash and sulphur content, with maturity ranging from lignite to high volatile bituminous [11]. Mega scopically, the coal beds are generally thinly banded (bands > 3–5 feet thick) and characterized by bright bands isolated in a matrix that is dominated by dull, resinous organic material. The chemical composition of coal in this region has a fixed carbon and ash content of 13.21% to 32.79%. The sulphur content is in the range of 5.45% to 10.63% with a moisture content of 3.14% to 4.26%. The proximate analysis for coal of the Salt Range is presented in the Punjab Mines and Minerals (PUNJMIN) results, as shown in Figure 2 [11]. 

**Figure 3:**
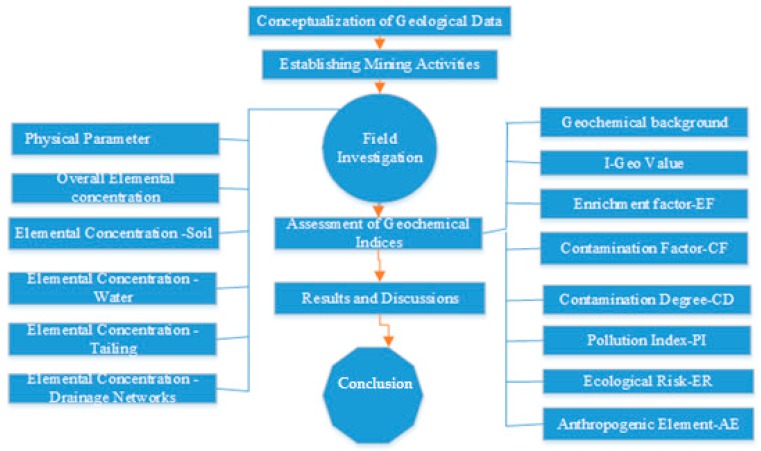
Framework for the assessment of physical parameters of water bodies near mining areas.

Figure 3 provides the detailed methodology of the present study. In the first phase, conceptual understanding of the geological data, stratigraphical units, strata dip, and strike were determined, along with the locations of mines and an accumulation of mine tailings. In the first phase of the objective, a cluster with more than 100 mines, located in the central Salt Range, was selected due to active mining activities. These mines are owned by the Punjab Mineral Development Company (PMDC), Punjab Mines and Minerals (PUNJMIN), and other local organizations. After establishing a route map and mining activities, samples of mine tailings, surface, and subsurface water and soil were collected by observing the critical approaches and standards.

### 2.2. Establishing Mining Activities

Shaft and the adit technique is, in practice, in the central Indus basin, in which the ground is excavated by manual tools up to a depth of the coal seam. On their way to the coal seam, they might intersect an aquifer, and puncture of the aquifer might cause a water inrush. A water inrush might lead to the flooding of the mines and a local depletion of groundwater [12]. This water inrush might lead to mine subsidence and, on the other hand, when this water is pumped out, mismanagement of this water might lead to open disposal to local tributaries, which travels to local ponds, like Chaab and Nilawahn, and ultimately to larger water bodies, such as streams and rivers. Approximately 4320 gallons per day of mine water is pumped and then flushed to the surface water resources of the area. This pumping is the reason for groundwater depletion, and the mixing of effluents causes different diseases among miners and the local community.

### 2.3. Field Investigationand Sampling Strategy

The main focus of the study was to determine the presence of potential toxic elements (PTEs) in water bodies. An atomic absorption spectrometer was used for the assessment of iron (Fe), cadmium (Cd), copper (Cu), chromium (Cr), nickel (Ni), manganese (Mn), zinc (Zn), lead (pb), and mercury (Hg). While using the random technique, a total of 50 samples from 12 selected sites of the mining areas, comprising of 10 soil samples, 10 stream sediment samples, 12 tailings samples, and 18 samples of surface and groundwater were collected in the months of May–June 2016–2018. The soil samples were collected with an auger tool from a depth of 80–100 cm, while stream sediment samples were taken by digging pits 0.5–1 m deep to have a bulk of 30kg 

### 2.4. Analytical Standards and Aspects

All of the samples were marked and packed in sealed plastic bags and transported for laboratory testing and analysis. After air drying (at 25 ± 2 °C), the samples were crushed by a jaw crusher to the size of −10 mm, reduced to −2 mm by pulverizing, and then finally −125 mm to have powder-like grains by a Tema mill [13]. Serial dilutions of every metal were made to 1000 mg/L solution by known standard stock solutions. Whenever required, the solutions were diluted, appropriately. Triplicate measurements were obtained and for quantification of selected minerals, the calibration line method was used. [14]. 

**Figure 4:**
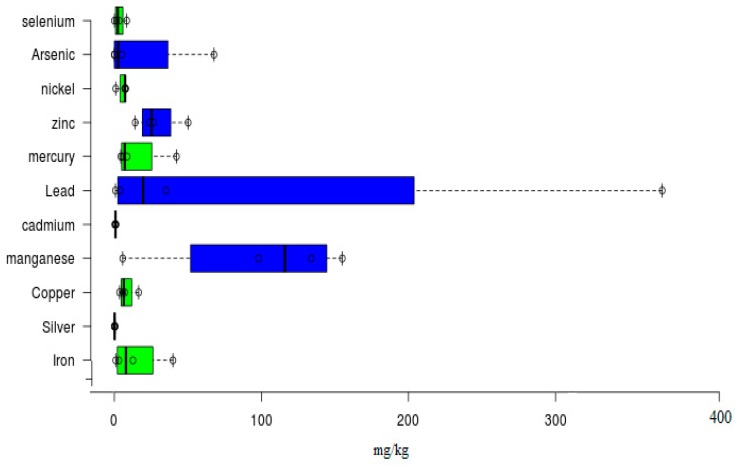
Estimated geological background elemental concentrations.

After air drying, tailings and soil and stream sediment were crushed to have a fine powder. Conventional aquaregia solutions were performed in 250 ml Teflon beakers. A well-mixed sample of 0.5 g of the sieved sediment soil or tailing) was digested in 12 ml of freshly prepared aqua-regia solution (1:3 HNO3–HCl, v/v) on a hotplate for 3 h at 110 °C. The solution was air-dried. The sample was diluted with aqueous nitric acid (20 mL, 2%), filtered through Whatman No. 42 paper into a 100 ml volumetric flask, and then diluted to 100 ml with deionized distilled water [15]. Violante and Adamo methods were used for the determination of pH [16]. Finally, the determination of selected PTEs, such as Zn, Cr, Ni, Cu, Pb, Mn, Cd, Hg, and Fe were made while using a Savant AA 5th generation of GBC Scientific Equipment Pty Ltd [17]. The air acetylene flame was calibrated by reading the manufacturer guidelines. Microsoft Excel 2013 and statistic software SPSS version 24 (SPSS Inc., Chicago, IL, USA) was used for making calculations. Later on, the results were also recalculated and retrofit by ANOVA statistical techniques, see Figure 4.

### 2.5. Assessment of Geochemical Indices

Before establishing geochemical indices, it is necessary to have geological or geochemical background values, to statically ascertain analysis and indices. After the analytical study, lab testing of rock and soil samples in virgin states, and comparison with available world data, established certain reference values for pertinent determination of geochemical indices. Figure 4 shows the calculated values, which have been expressed by Box and Whisker plots, and developed by statistical tools ANOVA.

The geo-accumulation index (I-geo), factor of enrichment (EF), factor of contamination (CF), and degree of contamination (CD) have been calculated, as defined by [18,19,20]. All of these geochemical indices of contamination have been calculated with respect to the calculated geological background, i.e., median values of metal concentration in samples of the study area. All of these geochemical indices are calculated by using the following equations:

I-geo=log2[Cn/1.5Bn]

EF=(Cn/Fe)sediment/(Cn/Fe,reference)

Cf=Cn/Bn

CD = The sum of all contamination factors (Cf)

$$\mathrm{PLI}=n\sqrt{\mathrm{CF}1\times \mathrm{CF}2\times \mathrm{CF}3\times \ldots .\mathrm{CFn}}$$

ER = Tr × Cf

$$\left[\frac{Cn-Bn}{Cn}\right]\times 100$$

$$PLI=n\sqrt{CF1\times CF2\times CF3\times \ldots .CFn}$$

In the above formulae, Cn is the concentration of the examined element ‘n’ in the surface sediments, Bn is the geochemical background concentration of metal ‘n’, and *Tr* is metal toxicity. The calculated surface rock average, as shown in Figure 4, has been used as a reference value of background concentrations in this work. The factor 1.5 in the I-geo formula (1) is incorporated to account for possible variation in the background data due to the lithologic effect [18]. The EF geochemical normalization equation (2), was obtained while using Fe as the reference element and as a conservative tracer to differentiate natural from anthropogenic components, following the hypothesis that its content in the earth crust has not been troubled by anthropogenic activity and because natural sources (98%) greatly dominate its contribution [21].

#### 2.5.1. Geo-Accumulation Index Value

The geo-accumulation index value that was developed by [18] is a popular tool for assessing metal concentration and possible pollution in the aquatic, terrestrial, and marine ecological units of any environment [18]. Geo-accumulation has five categories of pollution level (1–5) i.e., unpolluted to highly polluted, see Table 1.

#### 2.5.2. Enrichment Factor (EF)

The factor of enrichment (EF) is found by comparison of each metal accumulation with any reference metal concentration; generally, Fe, Al, and Mn, are well known reference metals [22]. Fe has been used as a conservative tracer to differentiate anthropogenic elements. The criteria for a factor of enrichment (EF) for PTEs, as shown in Table 2, and is found as a ratio of elemental concentration of sediment normalized to Fe [18]: The enrichment factor (EF) consists of categories (0–5) that range from depleted or minimum enrichment to extremely high enrichment > 40.

#### 2.5.3. Contamination Factor (CF) and Degree of Contamination (CD) 

The factor of contamination (CF) is described as the ratio of concentration in the tested sample to the reference geochemical background values of that element [20]. Similarly, the degree of contamination (CD) is described as the sum of all contamination factor values for PTEs [23]. The factor of contamination (CF) is defined according to four categories, given, as follows: <1 = low contamination factor, 1–3 = moderate contamination factors, 3–6 = considerable contamination factors, and >6 = very high contamination factor [24,25,26]. The degree of contamination (CD) is the sum of CFs. It allows for the assessment of the polymetallic contamination for each sampling site [27,28]. The degree of contamination is categorized as: <8 = low degree of contamination, 8–16 = moderate degree of contamination, 16–32 = considerable degree of contamination, and >32 = very high degree of contamination [25].

#### 2.5.4. Pollution Load Index (PLI)

The Pollution Load Index is a good indicator for assessing the pollution effects of any area and for any site, it is defined as follows: Greater than 1 value of PLI, is considered as being polluted and less than 1 is considered as no pollutant [28,29].

#### 2.5.5. Ecological Risk 

The Potential Ecological Risk Index (PERI) was proposed by [20] to be used as a diagnostic tool for ecological pollution. The potential ecological risk index method [20] uses the degree of accumulation of PTEs in sediment relative to the highest background value in sediment before industrialization and the corresponding ecological toxicity coefficient, with a weighted sum leading to the ecological hazard index [30]. Table 3 presents the criteria for the degree of ecological risk.

#### 2.5.6. Anthropogenic Element (AE) 

The potential anthropogenic elements affecting the ecology of coal mining areas, as criteria, AE >40 is considered to be a potential minimum affecting element [31,32]. In this case, at present, iron and copper are identified as the most prominent anthropogenic elements, rather than from natural sources of the area.

## 3. Results and Discussion 

### 3.1. Overall Elemental Concentration and Physical Parameters

**Figure 5:**
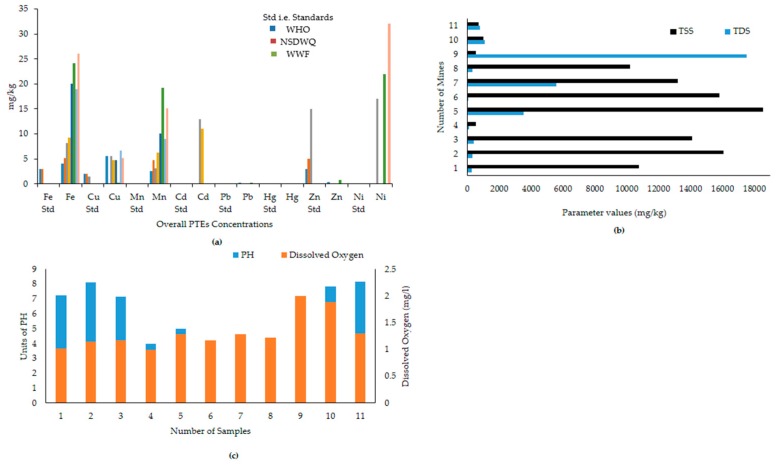
Overall concentration of potential toxic elements (PTEs) and the physical parameters in the study area.

This study is the outcome of research conducted from 2016 to 2018 and it is based on fieldwork, lab work, and analysis of the obtained results. Figure 5a shows the PTE concentrations in the years 2015–2016 and 2017–2018. It is evident that PTEs have been found, and some of them are beyond the acceptable limits of the WHO and NSDWQ standards. The presence of the following PTEs has been revealed, as follows: Ni > Fe > Mn > Zn > Cd > Cu > Pb > Hg. Likewise, physical parameters have also been developed, as in Figure 5b. The parameters analyzed included dissolved oxygen (DO), pH (Figure 5c), total dissolved solids (TDS), and total suspended solids (TSS).

**Figure 6:**
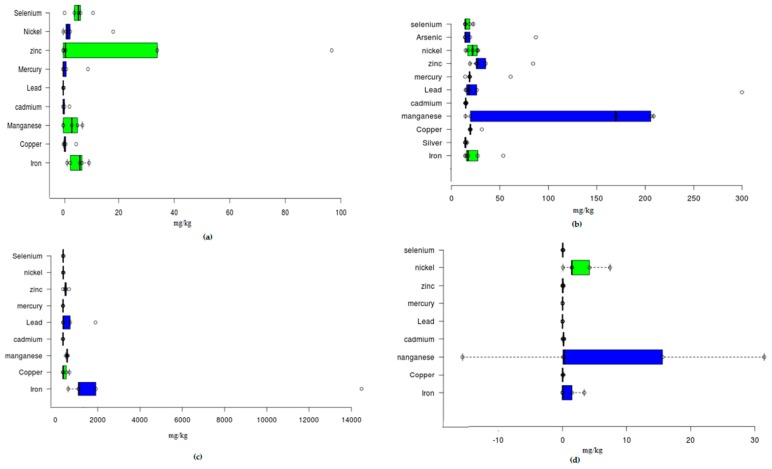
Box and Whisker plot for overall concentration of PTEs in the soil (**a**), water (**b**), tailings (**c**), and River Jhelum (**d**).

Figure 6 shows the overall PTEs concentration in the surrounding soil, tailing, and water bodies of the study area during the dry season in a statistical approach. Box and Whisker plots have also been established to assess the mean concentration with related statistics parameters and values. The order of increasing concentration has been established, as follows: for Fe: Tailings > Soils > Drainage networks > Mine water. For Mn: Mine water > Tailings > Jhelum River Drainage networks > Soils. For Zn; Soils > Tailings > Mine water > Drainage networks. For Pb: Mine water > Tailings > Drainage networks > Soils. For Hg: Soils > Mine water > Tailings > Drainage networks. The obtained results show that these concentration levels are high enough for available standards, like WHO, NSDWQ, and the Canadian Soil Quality Guidelines. These results are verified by the determination of available geochemical indices, like I-Geo (Geo-accumulation index), EF (Enrichment Factor), and contamination factor (CF), and contamination degree (CD). 

**Figure 7:**
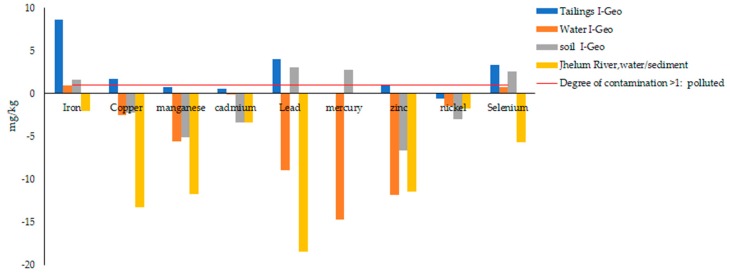
I-Geo value for overall concentration of PTEs in the study area.

Based on Figure 7, the calculated I-Geo values indicate that the River Jhelum were unpolluted, with values for iron, copper, manganese, lead, mercury, zinc, cadmium, nickel, and selenium with I- Geo value ≤ 0; and, unpolluted to moderately polluted or enriched for iron manganese, cadmium, and selenium in the case of tailings and mine water, where they all have I-Geo = 0–1. Mine water is unpolluted to moderately polluted by Fe and Se with order; Fe > Se. soils are strongly polluted by order; Pb > Hg > Se > Fe. The tailings sediments are polluted by the order; Fe > Pb > Se > Cu. Based on mean I-Geo values, it is anticipated that the central Indus Basin is strongly affected by PTEs in the order: Fe > Pb > Se > Hg > Cu.

**Figure 8:**
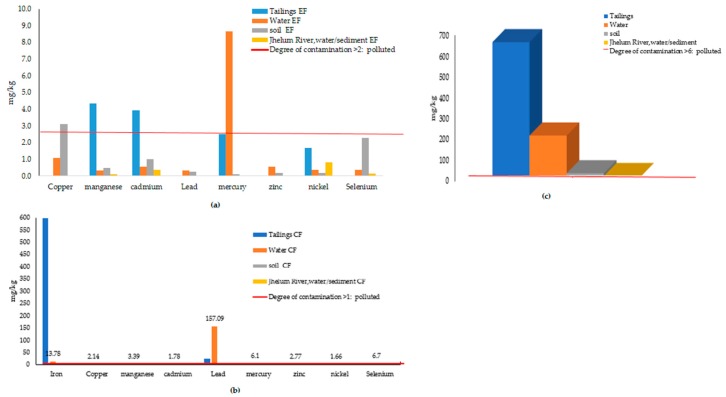
Geo-chemical indices for overall concentration of PTEs in the study area.

Figure 8a shows a factor of enrichment, which ranges from minimal enrichment to significant enrichment. River Jhelum is merely enriched by PTEs and all elements lies below minimum threshold value of EF (see Table 2). Cu and Se are enriching soils of the Central Indus Basin, but Cd is also seen on half of permissible limit of EF. The order of mean enrichment is found as follows: Hg > Mn > CD > Se > Ni > Zn. Figure 8b shows the results of the contamination factor in the Central Indus Basin, which indicates that the soils of the study area have low levels of contamination for iron, manganese, cadmium, zinc, nickel, lead, mercury, and selenium, while mine water are moderately contaminated by lead, iron, and selenium. However, the values are very high in the case of tailings, especially for iron and lead. Likewise, the results of the contamination degree (CD), Figure 8c, indicate that the Central Indus basin has a very high degree of contamination by anthropogenic pollution input, especially by tailings and mine water.

**Figure 9:**
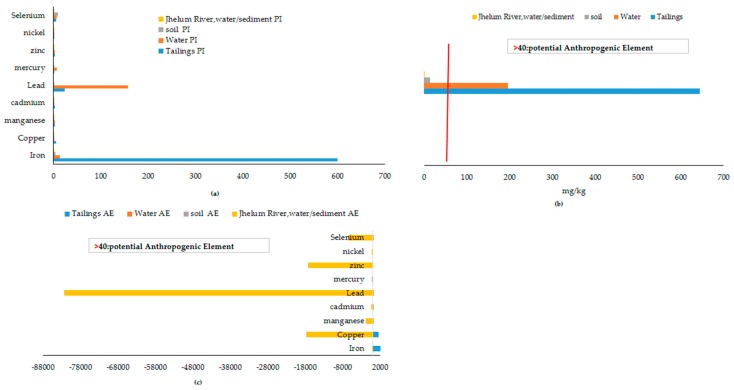
Pollution index and ecological risk indices for the overall concentration.

Figure 9a suggests the pollution index (PI), as follows: Tailings > Mine water > Soil, while the pollution index for River Jhelum is low to moderate. Figure 9b indicates the calculated results of the potential ecological risk (ER) are as follows; Tailings > Mine water, while the soil ecological risk lies below the threshold value of 40.In the case of the anthropogenic elements, at present, iron and copper are identified as the most prominent anthropogenic elements, rather than natural sources of the area (Figure 9c). Furthermore, Mn, Se, Cd, and Zn are emerging anthropic loads.

**Figure 10:**
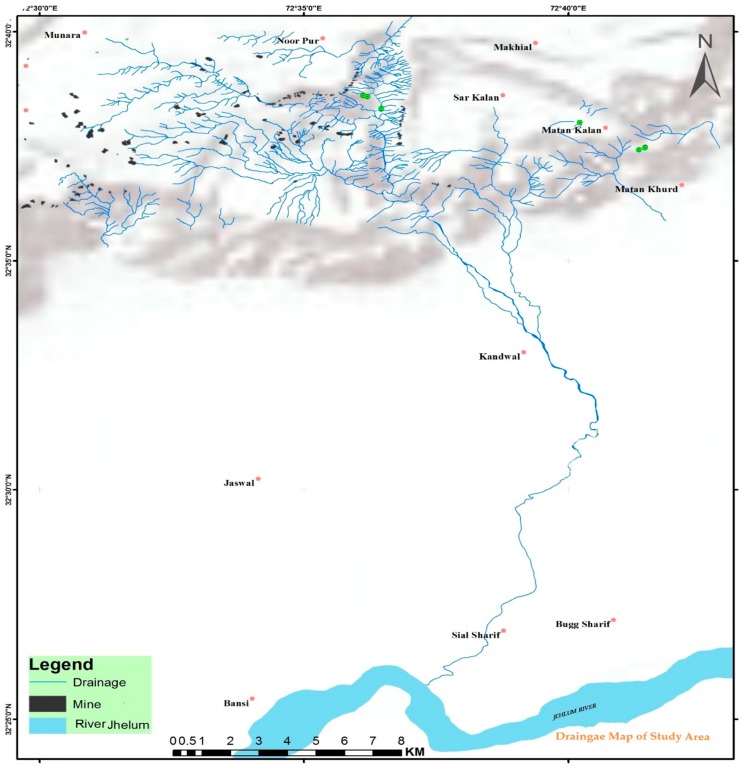
Linked drainage, ultimately mobilizing PTEs to main River Jhelum (Source Digital Elevation Model ASTER).

All of the calculated results were confirmed by the mineral composition of the surrounding rock and soil. Further confirmation was also made by the assessment of the geochemical indices, and rational values were calculated and presented in this study. The upstream pond of Neela Wahn (local recreational pond) shows the neutral level of contamination and it is considered fit for recreational and other usable purposes, but, at the downstream side, acid mine drainage (AMD) from mines is being added and is degrading the natural aesthetic of the water and soil, and ultimately the PTE load is affecting the main river body of the Central Indus Basin i.e., River Jhelum, as shown in Figure 10. A similar study that was conducted on the River Indus also supports our findings, as a concentration of PTEs is also found at River Indus Basin monitoring stations, where they ultimately drain to the Arabian Sea [28].

The mean, range, and standard deviation values of PTEs estimated in Central Indus Basin were collected (see Figure 5a). In addition, percentile values (10th, 25th, 50th, 75th, and 90th) of PTEs levels were analyzed (see Figure 6) and the percentages of mining areas in comparison with available standards (TEC and PEC) were calculated in order to substantiate the comparisons with standards (Table 4). Table 4 and Table 5 show the comparison of present study by mean values with finding of PTEs in other parts of world. The reported results of this study have been compared with the corresponding sediment quality guidelines (SQGs) [33,34].

Sediments of local tributaries of River Jhelum are highly contaminated with Iron as tailing sediments has concentration levels up to 898.08 mg/kg, no study is available for the river sediments of this region. Mn is second emerging element with elemental concentration of 430.9 mg/kg, while others areas of the Indus basin e.g. Marala, Qadirabad also have a significant concentration of 375 mg/kg and 319 mg/kg [35]. No study is available in the case of Mn for other areas of Asia. Cd, a well carcinogenic element, has higher concentrations, like tailings have 3.52 mg/kg and soils have 7.33 mg/kg, while others areas of the Indus basin have lower concentrations of 0.4 mg/kg and 0.3 mg/kg [35]. A higher concentration is comparable with neighboring countries like China (11.0 mg/kg) [36,37] and India (3.82 mg/kg) [38,39]. Sediments of Spain areas also show higher concentration of 6.59 mg/kg [42,43]. Ni with 24.56 mg/kg is the fourth prominent element, reaching SQGs [33,34]. A similar upraise in concentration is observed in China (29.1 mg/kg) [36,37] and India (83.7 mg/kg) [38,39]. Afterwards, Se (27 mg/kg) is an emerging element, especially in tailings and soils, which is comparable with Iran (23 mg/kg) [40,41], Spain (13mg/kg), and Vietnam (12mg/kg) [46,47]. 

Moreover, the water quality comparison for River Jhelum water has been established with other famous rivers of the world (Table 5), which reveals the lower contamination levels as compared to other rivers, but with increasing industrialization and unorganized mining activities, especially tailings, abandoned mines pose severe threats of increased contamination. A similar study on river Ravi, another prominent river of the Indus Basin, has reported the extinction of carp species, like Catla, Labeo rohita, and Cirrhinamrigala, due to increased contamination of PTEs, which might create adverse growth conditions in the concerned river and ecological integrities [56,57].

The overall level of toxicity as compared to other regional data (Table 4 and Table 5), contamination appears to be on lower side, which is due to the small scale of mining, lower discharges of AMD, and under-developed techniques of mining. However, some values, like Iron, Mn, Ni, Cd, and Se, are above or near the SQGs, which prognosticate the fate of the Central Indus Basin on the verge of under stress ecological status. 

On the basis of geochemical tools, like I-geo values, the toxicity of sediment is determined as Tailings sediments > mine water > soil sediments > River Jhelum, and elemental order of toxicity on the basis of PTEs is: Fe > Pb > Se > Hg > Cu. On basis of this study, Iron and Copper are designated as anthropogenic elements that need to be arrested by suitable remedial techniques. Furthermore, Mn, Cd, Hg, Pb, and Se, respectively, are emerging PTEs, which should also be the part of action plan.

The outcomes of this study will fill the knowledge gap for this part of Asia and will serve as the data set for comparison of toxicity, anthropic loads, pressure sources, and vulnerability scenarios to ensure ecological security and river resilience of Central Indus Basin.

### 3.2. Probable Toxicity and Health Effects of PTEs

**Figure 11:**
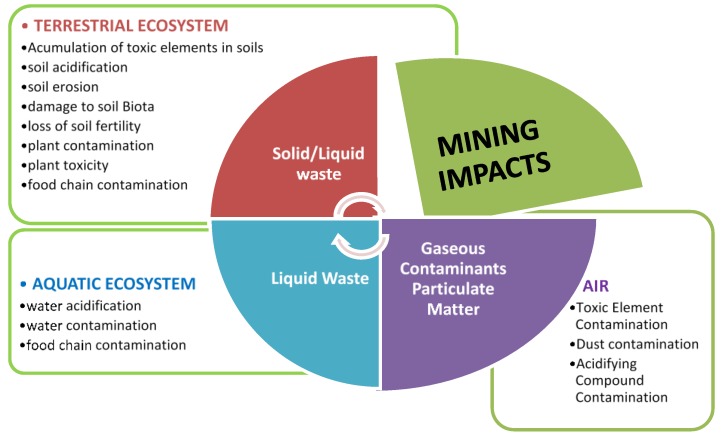
Potential environmental effects of the mining industry.

Mining anthropogenic effects have always been a serious concern for ecological resources of leased areas. The consequences of mining activities affect every element of the environment, as in Figure 11. Mine waters have a tendency, even with a neutral or alkaline nature, to dissolve PTEs, if in excess [50]. Active mining areas have a higher concentration of PTEs in the water, which is a serious menace to the health of the local habitants.

The pH and TDS of the surface water of local recreational water bodies and large water bodies show neutral to slightly alkaline pH nature and compliance with Pakistan’s Government and WHO standards (Figure 5b,c). Some areas around the mines have serious acid mine drainage issues with the potential to degrade larger water bodies of the Central Indus Basin, especially the River Jhelum. Likewise, excessive TDS can trigger eutrophication, ultimately disturbing the aesthetic structure of the water ecology of ponds. Moreover, excessive TDS around mines as unmatchable with WWF standards for recreational water. It can be anticipated by geochemical indices, the main influencing factors of water pollution in the Central Indus Basin are regarded as being anthropogenic. 

In general, the toxicity of metal ions to mammalians systems is due to chemical reactivity of the ions with cellular structural proteins, enzymes, and membrane system. This is often dependent on the route of exposure and the chemical compound of the metal i.e. its valiancy state, volatility, lipid solubility, etc. PTEs may exert their acute and chronic effects on the human skin through stress signals [58]. Based on literatures, the probable toxic health effects of prominent PTEs found in this study have been discussed.

Iron poisoning has been of keen interest for medical researchers, especially in infants and children while using a food supplement [59]. Iron up to 26–36 mg/kg has been found in mine water. Iron is a very common and essential part of our food chain, as it is a cofactor in many enzymes and proteins. Miners that are exposed to free intracellular iron may have DNA damage, and iron can cause cancer by oxidation of DNA molecules. This might cause health problems, like hypotension, shocks, lethargy, hepatic necrosis, tachycardia, metabolic acidosis DNA damage, and ultimately death. The oxidation of ferrous (Fe^2+^) to ferric (Fe^3+^) cations and the presence of Thiobacillus ferroxidants bacteria produces enough acidity to eventually cause dissolving and mobility of PTEs to distant locations [60]. 

The highest lead concentrations were found up to 0.28 mg/kg, and lead has been a major cause of diseases that are associated with the central nervous system, gastrointestinal tract, and mental abnormalities, psychosis, autism, allergies, paralysis, dyslexia, brain, and kidney damage, and in severe cases to death [61]. Nickel, up to a maximum of 32 mg/kg, has been found in mine water, and it might cause depletion of glutathione and cause DNA modifications, lipid peroxidation, and sulfhydryl homeostasis [62].

Cadmium, up to 13 mg/kg, has been found in one mine. Cadmium is notorious for its toxic damages to the kidneys and its accumulation in the proximal tubular cells in chronic exposure, and severe damage to the human skeletal system, along with bone mineralization [63]. There are two genetic disorders regarding copper, Wilson’s disease (WD) and Menkes Disease (MD), which result from mutations in enzymes that are involved in the transport of copper into cells of the body and IHD accompanied with bone and joint pain, chest pain, along with complaints of frequent backache, abdominal pain, and browning of hair problems [63]. Other metals like zinc, mercury, and chromium, were found below the permissible limits of available standards. However, increasing mining activities and abandoned mines may have serious contamination spills and might cause irreparable damages to water resources of the area and pose serious threats to human health and the environment. To avoid health issues regarding PTEs, contaminated soil might require soil removal, excavation, burial, and cleaning treatment processes, like soil washing and flushing [64].

## 4. Conclusions

There are no specific studies regarding the Central Indus Basin in the context of PTE pollution. Therefore, geochemical background reference values were calculated to find natural water and soil contamination and differentiate the anthropogenic contribution of mining activities. Geochemically, the formation of AMD is a complex process and it is influenced by the function of microbiological control, the depositional environment, and acid/base balance of tailing, basin morphology, inferred lithology, mineralogy, and hydrological conditions of the area, so all of these parameters need to be considered while developing pollution indexes.

Based on I-geo values, tailings sediment and mine water are found main sources of toxicity, threatening the good ecological status of Central Indus Basin. Iron and Copper are found as anthropic loads with Mn, Cd, Hg, and Se, as emerging toxic elements. Despite its limitations, this study gives the first description of the overall pollution levels and health risks that are posed by heavy metals in ecology of various mining areas throughout Central Indus Basin, Pakistan.

We conclude that higher PTEs content in the water is assumed to be anthropogenic rather than natural, as the area is well known for coal and other mining activities. This poses a severe threat to the water ecology of the area. Moreover, rainwater can transport these PTEs to lower agriculture lands and, ultimately, drain to the main water body of the Central Indus Basin and the River Jhelum. Proper management of mine’s tailings should be mandatory by the concerned organization, as they are a potential source of toxic trace elements in the local ecosystem of the Central Indus Basin. Regular monitoring of PTEs in water quality is recommended around the active and abandoned mining sites for the conservation and protection plan of ecological resources.

We recommend the regulation of the public mining in the Central Indus Basin, along with a rehabilitation plan, and miners should be provided with proper training and education on the provision of environmental protection, especially on the management of mine’s tailings and its runoff to the natural environment.

The outcomes of this study will help to create an environmental database, and concerned authorities will be able to monitor the pollution and suggest suitable developments to control the transport of PTEs in the local soil, rock, and water bodies of the Central Indus Basin.

## Figures and Tables

**Table 1:** Geo-accumulation index scale.

I-Geo Value	Pollution Value
<0	Unpolluted
0–1	Unpolluted to Moderately polluted
1–2	Moderately polluted to Strongly polluted
2–3	Strongly polluted
3–5	Strongly polluted to extremely strongly polluted
>5	Extremely polluted

**Table 2:** Enrichment factor scale.

Enrichment Factor (EF)	Description
EF < 2	Depletion to minimal enrichment
2 < EF < 5	Moderate enrichment
5 < EF < 20	Significant enrichment
20 < EF < 40	Very high enrichment
EF > 40	Extremely high enrichment

**Table 3:** Ecological risk scale.

Range	Ecological Risk
<40	Low Ecological Risk
40 ≤ ER ≤ 80	Moderate potential Ecological Risk
80 ≤ ER ≤ 160	Considerable potential Ecological Risk
160 ≤ ER < 320	Highpotential Ecological Risk
ER > 320	Very high potential Ecological Risk

**Table 4:** Comparison of metal levels (mg/kg) in sediments and mine water, determined in this study, with those reported in the literature.

Mean Concentrations of PTEs (mg/kg)										
Location	Zn	Mn	Cu	Cd	Pb	Hg	Fe	Ni	Se	Reference
Tailing’s *	77.62	430.9	15.9	3.52	18.59	12.5	898.08	19	27	Present study
Soil *	28.8	274.89	5.6	7.33	0.021	0.001	19.5	4.2	16.08	
Marala	23	375	6	0.4	11.9	-	-	-	-	[35]
Qadirabad	22	319	5.4	0.3	4	-	-	-	-	[35]
china	1163	-	211.9	11	641.3	3.82	198	29.1	-	[36,37]
India	338.8	-	63.9	3.82	304.7	-	166.2	83.7	-	[38,39]
Iran	363.4	-	88.4	1.49	1002	3.13	50	1.8	23	[40,41]
Spain	465.8	-	120.8	6.59	881.8	52.9	294	13	13	[42,43]
South Korea	183.2	-	79.09	1.99	111.1	1.12	22.1	-	-	[44,45]
Vietnam	41.09	-	271.4	1.35	30635	-	3.7	20	12	[46,47]
TEC	121	460	31.6	0.99	35.8	-	-	22	-	[33]
PEC	459	110	149	4.98	128	-	-	48.6	-	[34]

* Represents the types of samples collected in study area.

**Table 5:** Comparison of PTEs levels (mg/kg) determined in this study, with those reported in the literature.

Location	Mean Concentration (mg/kg)							
	Zn	Mn	Cd	Cu	Co	Pb	Ni	Reference
River Jhelum, Pakistan	0.245	0.73	0.275	0.027	-	0.007	10.1	This study
River Chenab, Pakistan	33.7	494	1.67	8.16	7.95	18.1	-	[35]
River Ravi, Pakistan	-	-	3.17	159.79	18.53	-	-	[48]
River Indus, Pakistan	54.3	215	1.62	33.2	-	2.71	-	[28]
River Tigris, Turkey	1061	1682	7.9	2860	516	66	-	[49]
River Rimac, Peru	8076	-	31	796	24	2281	-	[50]
River Gomati, India	99.4	320	7.90	35.7	-	92.2	-	[51]
River Second Songhua, China	403	-	-	78.9	14.7	124	-	[52]
River Hindon, India	85	202	3.47	195	-	59.1	-	[53]
River Almendares, Cuba	709	-	43	421	-	189	-	[54]
River Nile, Egypt	221	2810	-	81	-	232	-	[55]

## References

[1] Prathap A., Chakraborty S. (2019). Hydro chemical characterization and suitability analysis of groundwater for domestic and irrigation uses in open cast coal mining areas of Charhi and Kuju, Jharkhand, India. Groundw. Sustain. Dev..

[2] Wellen C., Shatilla N.J., Carey S.K. (2018). The influence of mining on hydrology and solute transport in the Elk Valley, British Columbia, Canada. Environ. Res. Lett..

[3] Qiao W., Li W., Li T., Chang J., Wang Q. (2017). Effects of coal mining on shallow water resources in semiarid regions: A case study in the Shennan mining area, Shaanxi, ChinaChina. Mine Water Environ..

[4] Griffith M.B., Norton S.B., Alexander L.C., Pollard A.I., LeDuc S.D. (2012). The effects of mountaintop mines and valley fills on the physicochemical quality of stream ecosystems in the central Appalachians: A review. Sci. Total Environ..

[5] Ali M.M., Ali M.L., Islam M.S., Rahman M.Z. (2016). Preliminary assessment of heavy metals in water and sediment of Karnaphuli river, Bangladesh. Environ. Nanotechnol. Monit. Manag..

[6] Malkani M.S., Buzdar F., Zahid M. (2016). Coal resources of PakistanPakistan: New coalfields. Lasbela Univ. J. Sci. Technol..

[7] Howladar M.F. (2013). Coal mining impacts on water environs around the Barapukuria coal mining area, Dinajpur, Bangladesh. Environ. Earth Sci..

[8] Yeats R.S., Khan S.H., Akhtar M. (1984). Late quaternary deformation of the salt range of Pakistan. Geol. Soc. Am. Bull..

[9] Ahmad H., Ahmad A., Jan M.M. (2002). The medicinal plants of salt range. Online J. Biol. Sci.

[10] Iqbal M. Soil & Water Conservation Needs Assessment using Geospatial Techniques: A case Study of Potohar Region of Pakistan. Proceedings of the 4th International Conference on the Use of Space Technology for Water Management.

[11] Malkani M.S., Mahmood Z. (2017). Mineral resources of Pakistan: Provinces and basins wise. Geol. Surv. Pak. Mem..

[12] Burger Chakraborty L., Qureshi A., Vadenbo C., Hellweg S. (2013). Anthropogenic mercury flows in India and impacts of emission controls. Environ. Sci. Technol..

[13] American Society for Testing and Materials (2003). ASTM D422-63, Standard test method for particle-size analysis of soils. Annual Book of ASTM Standards.

[14] Radojevic M., Bashkin V. (2007). Practical Environmental Analysis.

[15] Gallego J.L.R., Ordóñez A., Loredo J. (2002). Investigation of trace element sources from an industrialized area (Avilés, northern Spain) using multivariate statistical methods. Environ. Int..

[16] Violante P., Adamo P. (2000). pH determination. Official Methods of Soil Chemical Analysis.

[17] USEPA (2001). Method 3051: Microwave Assisted Digestion of Sediments, Sludges, Soils and Oils, Official Methods/US EPA Methods.

[18] Muller G. (1969). Index of geoaccumulation in sediments of the Rhine river. Geojournal.

[19] Sutherland R. (2000). Bed sediment-associated trace metals in an urban stream, Oahu, Hawaii. Environ. Geol..

[20] Hakanson L. (1980). An ecological risk index for aquatic pollution control. A sedimentological approach. Water Res..

[21] Idris I.M., Younis Y.M.E., Elbashir A.A. Monitoring the anthropogenic and geochemical environment surrounding the Butana drinking water sources via the determination of heavy metals composition of the soil, streams sediments and gold mining tailings (ii). https://www.eijst.org.uk/images/frontImages/articles/Vol.7No.3/6.51-64.pdf.

[22] Liu W.-H., Zhao J.-Z., Ouyang Z.-Y., Söderlund L., Liu G.-H. (2005). Impacts of sewage irrigation on heavy metal distribution and contamination in Beijing, China. Environ. Int..

[23] AKSU A.E., YAŞAR D., Orhan U. (1998). Assessment of marine pollution in Izmir Bay: Heavy metal and organic compound concentrations in surficial sediments. Turk. J. Eng. Environ. Sciences.

[24] Likuku A.S., Mmolawa K.B., Gaboutloeloe G.K. (2013). Assessment of heavy metal enrichment and degree of contamination around the copper-nickel mine in the Selebi Phikwe region, Eastern Botswana. Environ. Ecol. Res..

[25] Rastmanesh F., Moore F., Kopaei M.K., Keshavarzi B., Behrouz M. (2011). Heavy metal enrichment of soil in sarcheshmeh copper complex, Kerman, Iran. Environ. Earth Sci..

[26] Sadhu K., Adhikari K., Gangopadhyay A. (2012). Assessment of heavy metal contamination of soils in and around open cast mines of Raniganj area, India. Int. J. Environ. Eng. Res..

[27] Halim M., Majumder R., Zaman M. (2015). Paddy soil heavy metal contamination and uptake in rice plants from the adjacent area of Barapukuria coal mine, northwest bangladesh. Arab. J. Geosci..

[28] Tariq J., Ashraf M., Jaffar M., Afzal M. (1996). Pollution status of the Indus river, Pakistan, through heavy metal and macronutrient contents of fish, sediment and water. Water Res..

[29] Krika A., Krika F. (2017). Evaluation of the status of heavy metal pollution in surface water and sediments of the Nile river (north eastern Algeria). Pollution.

[30] Li Z., Huo J., Bricker J.D. (2019). Ecological risk assessment for eutrophication and heavy metal pollution of suyahu reservoir sediments. Biotechnol. Biotechnol. Equip..

[31] Ebong G.A., Ekong C.I. (2015). Pollution status of trace metals in waste impacted soils within Borokiri town, Port Harcourt metropolis, Rivers state, Nigeria. Int. J. Sci. Res. Environ. Sci..

[32] Mazurek R., Kowalska J., Gąsiorek M., Zadrożny P., Józefowska A., Zaleski T., Kępka W., Tymczuk M., Orłowska K. (2017). Assessment of heavy metals contamination in surface layers of Roztocze National Park forest soils (se poland) by indices of pollution. Chemosphere.

[33] MacDonald D.D., Ingersoll C.G., Berger T.A. (2000). Development and evaluation of consensus-based sediment quality guidelines for freshwater ecosystems. Arch. Environ. Contam. Toxicol..

[34] Caeiro S., Costa M.H., Ramos T.B., Fernandes F., Silveira N., Coimbra A., Medeiros G., Painho M. (2005). Assessing heavy metal contamination in Sado Estuary sediment: An index analysis approach. Ecol. Indic..

[35] Hanif N., Eqani S.A.M.A.S., Ali S.M., Cincinelli A., Ali N., Katsoyiannis I.A., Tanveer Z.I., Bokhari H. (2016). Geo-accumulation and enrichment of trace metals in sediments and their associated risks in the Chenab river, Pakistan. J. Geochem. Explor..

[36] Yuan G.L., Sun T.H., Han P., Li J. (2013). Environmental geochemical mapping and multivariate geostatistical analysis of heavy metals in topsoils of a closed steel smelter: Capital Iron & Steel Factory, Beijing, China. J. Geochem. Explor..

[37] Duan J., Tan J. (2013). Atmospheric heavy metals and arsenic in China: Situation, sources and control policies. Atmos. Environ..

[38] Prasad B., Kumari P., Bano S., Kumari S. (2014). Ground water quality evaluation near mining area and development of heavy metal pollution index. Appl. Water Sci..

[39] Reza S.K., Baruah U., Singh S.K., Das T.H. (2015). Geostatistical and multivariate analysis of soil heavy metal contamination near coal mining area, Northeastern India. Environ. Earth Sci..

[40] Ghaderpoori M. (2018). Heavy metals analysis and quality assessment in drinking water–Khorramabad city, Iran. Data Brief.

[41] Pirsaheb M., Khosravi T., Sharafi K., Babajani L., Rezaei M. (2013). Measurement of heavy metals concentration in drinking water from source to consumption site in Kermanshah—Iran. World Appl. Sci. J..

[42] Alvarez E., Marcos M.F., Vaamonde C., Fernández-Sanjurjo M.J. (2003). Heavy metals in the dump of an abandoned mine in Galicia (NW Spain) and in the spontaneously occurring vegetation. Sci. Total Environ..

[43] Hudson-Edwards K.A., Schell C., Macklin M.G. (1999). Mineralogy and geochemistry of alluvium contaminated by metal mining in the Rio Tinto area, southwest Spain. Appl. Geochem..

[44] Kim K.K., Kim K.W., Kim J.Y., Kim I.S., Cheong Y.W., Min J.S. (2001). Characteristics of tailings from the closed metal mines as potential contamination source in South Korea. Environ. Geol..

[45] Lee J.Y., Choi J.C., Lee K.K. (2005). Variations in heavy metal contamination of stream water and groundwater affected by an abandoned lead–zinc mine in Korea. Environ. Geochem. Health..

[46] Thuy H.T., Tobschall H.J., An P.V. (2000). Distribution of heavy metals in urban soils–a case study of Danang-Hoian Area (Vietnam). Environ. Geol..

[47] Tra Ho T.L., Egashira K. (2000). Heavy metal characterization of river sediment in Hanoi, Vietnam. Commun. Soil Sci. Plant Anal..

[48] Abdul R., Muhammad J., Muhammad U., Sajid A. (2009). Assessment of heavy metals in sediments of the river ravi, Pakistan. Int. J. Agric. Biol..

[49] Varol M., Şen B. (2012). Assessment of nutrient and heavy metal contamination in surface water and sediments of the upper Tigris river, Turkey. Catena.

[50] Méndez W. (2005). Contamination of Rímac river basin Peru, due to mining tailings. Environmental Engineering and Sustainable Infrastructure.

[51] Singh V.K., Singh K.P., Mohan D. (2005). Status of heavy metals in water and bed sediments of river gomti–a tributary of the ganga river, India. Environ. Monit. Assess..

[52] Lin C., He M., Zhou Y., Guo W., Yang Z. (2008). Distribution and contamination assessment of heavy metals in sediment of the second Songhua river, China. Environ. Monit. Assess..

[53] Suthar S., Nema A.K., Chabukdhara M., Gupta S.K. (2009). Assessment of metals in water and sediments of Hindon river, India: Impact of industrial and urban discharges. J. Hazard. Mater..

[54] Olivares-Rieumont S., De la Rosa D., Lima L., Graham D.W., Katia D., Borroto J., Martínez F., Sánchez J. (2005). Assessment of heavy metal levels in Almendares river sediments—Havana city, Cuba. Water Res..

[55] Rifaat A. (2005). Major controls of some metals distribution in sediments off the Nile Delta, Egypt. Egypian J. Aquat. Res..

[56] Azmat H., Javed M., Jabeen G. (2012). Acute toxicity of aluminium to the fish (Catla catla, Labeo rohita and Cirrhina mrigala). Pak. Vet. J..

[57] Qadir A., Malik R.N. (2011). Heavy metals in eight edible fish species from two polluted tributaries (Aik and Palkhu) of the River Chenab, Pakistan. Biol. Trace Elem. Res..

[58] Singh R., Gautam N., Mishra A., Gupta R. (2011). Heavy metals and living systems: An overview. Indian J. Pharmacol..

[59] Chaturvedi R., Banerjee S., Chattopadhyay P., Bhattacharjee C.R., Raul P., Borah K. (2014). High iron accumulation in hair and nail of people living in iron affected areas of Assam, India. Ecotoxicol. Environ. Saf..

[60] Patil R.M., Thorat N.D., Shete P.B., Bedge P.A., Gavde S., Joshi M.G., Tofail S.A., Bohara R.A. (2018). Comprehensive cytotoxicity studies of superparamagnetic iron oxide nanoparticles. Biochem. Biophys. Rep..

[61] Cherfi A., Achour M., Cherfi M., Otmani S., Morsli A. (2015). Health risk assessment of heavy metals through consumption of vegetables irrigated with reclaimed urban wastewater in Algeria. Process Saf. Environ. Prot..

[62] Izah S.C., Chakrabarty N., Srivastav A.L. (2016). A review on heavy metal concentration in potable water sources in Nigeria: Human health effects and mitigating measures. Expo. Health.

[63] Chakraborty S., Dutta A.R., Sural S., Gupta D., Sen S. (2013). Ailing bones and failing kidneys: A case of chronic cadmium toxicity. Ann. Clin. Biochem..

[64] Race M., Ferraro A., Fabbricino M., La Marca A., Panico A., Spasiano D., Tognacchini A., Pirozzi F. (2018). Ethylenediamine-N, N′-disuccinic acid (EDDS)—Enhanced flushing optimization for contaminated agricultural soil remediation and assessment of prospective Cu and Zn transport. Int. J. Environ. Res. Public Health.

